# Damage from dissection is associated with reduced neuro-musclar transmission and gap junction coupling between circular muscle cells of guinea pig ileum, *in vitro*

**DOI:** 10.3389/fphys.2014.00319

**Published:** 2014-08-22

**Authors:** Simona E. Carbone, David A. Wattchow, Nick J. Spencer, Timothy J. Hibberd, Simon J. H. Brookes

**Affiliations:** ^1^Centre for Chronic Disease Prevention and Management, Enteric Neuropathy Laboratory, College of Health and Biomedicine, Victoria UniversityMelbourne, VIC, Australia; ^2^Neurogastroenterology Laboratory, Surgery, Discipline of Human Physiology and Centre for Neuroscience, Flinders Medical Science and Technology, Flinders UniversityAdelaide, SA, Australia; ^3^Discipline of Surgery and Centre for Neuroscience, Flinders UniversityAdelaide, SA, Australia

**Keywords:** damage, neurotransmission, gap junction coupling, smooth muscle, intestine

## Abstract

Excitatory and inhibitory junction potentials of circular smooth muscle cells in guinea pig ileum and colon are suppressed 30–90 min after setting up *in vitro* preparations. We have previously shown this “unresponsive” period is associated with a transient loss of dye coupling between smooth muscle cells, which subsequently recovers over the ensuing 30–90 min; junction potentials recover in parallel with dye coupling (Carbone et al., 2012). Here, we investigated which components of dissection trigger the initial loss of coupling. Intracellular recordings were made from circular muscle cells of guinea pig ileum with micropipettes containing 5% carboxyfluorescein. After allowing 90–120 min for junction potentials to reach full amplitude, we re-cut all 4 edges of the preparation more than 1 mm from the recording sites. This caused a reduction in the amplitude of IJPs from 17.2 ± 0.7 mV to 9.5 ± 1.5 mV (*P* < 0.001, *n* = 12) and a significant reduction in dye coupling. Both recovered within 60 min. We repeated this experiment (*n* = 4), recording both 1 and 4 mm from the cut edge: both sites were equally affected by re-cutting the sides of the preparation. Equilibrated preparations were stretched to 150% of their original length, this had no significant effect on junction potentials or dye coupling. Setting up preparations in low calcium solution did not prevent the initial suppression of IJPs and dye coupling. Application of 3 μM indomethacin (*n* = 3), 10 μM ketotifen (*n* = 4) or 10 μM forskolin during dissection did not prevent the suppression of IJPs and dye coupling. If dissection damage was reduced, by leaving the mucosa and submucosa attached to the circular muscle, IJPs showed less initial suppression than in preparations where the layers were dissected off. We conclude that physical damage to the gut wall triggers loss of gap junction coupling and neuromuscular transmission, this is not due to stretch, influx of calcium ions, release of prostaglandins or mast cell degranulation. The mechanisms underlying this potent effect remain to be determined.

## Introduction

Mixing and propulsion of content along the gastrointestinal tract result from an organized series of contractions and relaxations of smooth muscle cells in the gut wall. Smooth muscle contraction is largely dependent upon membrane potential; depolarisation above a threshold causes contraction. Smooth muscle cells in most regions of gut are electrically and metabolically coupled to each other by gap junctions. Slow waves, initiated by interstitial cells of Cajal (ICC) rhythmically depolarise the smooth muscle syncytium (Dickens et al., 1999; Huizinga and Lammers, 2008; Sanders et al., 2012). Excitatory neurotransmitters from enteric motorneurons further depolarise the smooth muscle (Gillespie and Mack, 1964), and if the threshold is reached, smooth muscle action potentials cause an influx of calcium ions which drive contraction. Transmitters from inhibitory motorneurons cause hyperpolarization (Bülbring and Tomita, 1967), which shift membrane potential away from threshold and directly relax the muscle. Smooth muscle cells, ICC and enteric nerve cells express a wide range of receptors, making these preparations ideal for a wide range of pharmacological studies, since the 1950s (Paton, 1955).

Following the initial setup of smooth muscle preparations *in vitro*, there is often a period in which preparations develop little contractile tone and fail to respond to electrical or pharmacological stimulation. Typically, experimenters allow 30–90 min for preparations to “equilibrate” or “warm-up” before commencing recordings. It has recently been shown in guinea pig small and large intestine that this initial unresponsive period is closely associated with reduced gap junction coupling between smooth muscle cells (Carbone et al., 2012). As gap junction coupling recovers over the “equilibration period,” responses to electrical stimulation of nerve fibers and to pharmacological agonists are restored. It is likely that gap junction coupling is actually involved in modulating responsiveness because pharmacological inhibition of gap junction coupling (by carbenoxolone or glycerrhitinic acid) causes a temporary suppression of junction potentials.

The initial suppression of excitability of smooth muscle is profound. The muscle is unable to respond to most types of stimulation during this period. Despite the potency of this effect, what triggers the loss of coupling has not been identified. When isolated preparations of smooth muscle are set up *in vitro*, they are subject to changes in temperature, stretch and cellular damage. In this study we investigated which of these factors contributes to the loss of responsiveness and gap junction coupling shown by smooth muscle preparations.

## Methods

Adult male guinea pigs were stunned by a blow to the back of the head and exsanguinated in a manner approved by the Animal Welfare Committee of Flinders University (South Australia). Segments of ileum at least 10 cm proximal to the cecum were removed from the animal and placed into cooled modified Krebs solution at 15°C. Preparations were dissected in Sylgard (Dow Corning, Midland, MI)-lined petri dish in Krebs solution at this temperature. Modified Krebs solution consisted of the following (mM): NaCl 118; KCl 4.70, NaH_2_PO_4_2H_2_O 1; NaHCO_3_25; MgCl_2._6H_2_O 1.2; D-Glucose 11; CaCl_2._2H_2_O 2.5; bubbled with 95%O_2_ and 5%CO_2_. The addition of 1 μM hyoscine and 1 μM nicardipine was used to inhibit excitatory junction potentials and muscle contractions. The segment of ileum was opened longitudinally along the mesenteric border and, unless otherwise specified, the mucosa and submucosa were removed by sharp dissection. The specimen was then cut down to a length of 10 mm, and the full circumference, of 8–10 mm, was maintained and it was re-pinned in a Sylgard lined recording chamber (3 ml volume) with 50 μm tungsten pins, with the circular muscle layer uppermost.

### Intracellular electrophysiology

The recording chamber was mounted on an inverted microscope (Olympus IX71, Olympus, Tokyo, Japan) with LED-array fluorescent optics (CoolLED, Andover, UK) and superfused with warmed Krebs solution so that the bath temperature stabilized at 35°C Time zero (*t* = 0 min) was defined as the moment at which warmed Krebs solution first reached the recording chamber. Circular muscle cells were impaled with borosilicate micropipettes (1 mm OD, 0.58 mm ID; Harvard Apparatus) filled with 1 mM KCl and 5% 5,6-carboxyfluorescein in 20 mM Tris buffer solution (pH 7.0, Sigma Aldrich, Sydney, Australia), with resistances of 50–150 MΩ. Conventional intracellular recordings were made via an Axoclamp 2A amplifier (Axon Instruments) and viewed on an oscilloscope (model VP-5220A). Signals were digitized at 10 kHz and recorded (MacLab 8SP, ADInstruments, Sydney, Australia) using Chart 7 software (ADInstruments) on a MacBook Pro computer. A Grass S48 stimulator and S1U5 stimulator isolation unit were used to deliver 15 V supramaximal stimuli via 50 μm Pt/Ir electrodes, insulated to within 100 μm of their tips, placed 1 mm directly circumferential to the recording site. Fast inhibitory junction potentials (IJPs), driven by single shot stimuli, were recorded at intervals from time zero (*t* = 0 min), with a minimum of 20 s interval between stimuli (Carbone et al., 2012). In some cases, 10 mM ATP (dissolved in Krebs solution) was applied directly to the impaled cell from a micropipette with 10 μm tip, with 20–50 ms pulses of nitrogen gas at 140 kPa pulses of N_2_ at 20–50 ms duration. The solution contained 0.1% inert blue food dye (Rainbow Food Dyes, Australia) to monitor the trajectory of the ejected fluid and the tip was placed ~100 μm from the recording site. Input resistance was estimated from intracellular current pulses as an electrical measure of coupling between smooth muscle cells. At the end of the recording, 0.5 nA hyperpolarising current, (0.2 s duration at 2.5 Hz for 2 min) were used to inject carboxyfluorescein into the recorded smooth muscle cell. The electrode was withdrawn and 1 min later the total number of carboxyfluorescein labeled cells was counted under a 10x objective, *in situ*, using standardized illumination intensity.

### Mechanisms that may trigger the unresponsive period

Previous studies have shown that cooling preparations to 15°C does not evoke an unresponsive period (Carbone et al., 2012). To test the effects of cutting through the smooth muscle layer, two rows of tungsten pins 1 mm apart were placed around the entire circumference of the tissue (*n* = 4). Recordings were made for 120 min after setting up this preparation. Then all 4 edges were re-cut between the rows of pins and recordings were again made. In all cases, impaled smooth muscle cells were more at least 2–3 mm from cut edges (i.e., at least 2 cell-lengths, or a quarter of the diameter of the specimen to ensure that physically damaged cells were not recorded). In a separate 4 preparations, the recovery period of cells was measured 1 vs. 4 mm from one side of the tissue (running oral—anal), the preparation was re-cut and the smooth muscle cells were impaled exactly 1 or 4 mm from the newly cut edge. Thirdly, the study was independently replicated by a different experimenter (TJH, *n* = 5), and the initial warm-up period was recorded, followed by the effects of re-cutting the side of the preparation. The effects of circumferential stretch (an unavoidable component of dissection) were studied as follows. An array of hooks, Brookes et al. (1999) was attached along one mesenteric edge of the preparation and attached to a micromanipulator. Smooth muscle cells were recorded in a fully equilibrated preparation, which was then stretched to 115, 130, and 150% of its original resting circumference for 5 min, then returned to resting length (100%). Further recordings were then compared to determine if they had changed junction potentials and dye coupling.

To test whether Ca^2+^ influx from the Krebs solution might contribute to the initial loss of responsiveness, another set of preparations (*n* = 4) were dissected in low [Ca^2+^], high [Mg^2+^] Krebs solution (0.25 and 10 mM respectively), then transferred to normal Krebs solution for recording. The time at which the preparations were first superfused with normal Krebs solution at 35°C was monitored and considered as time = 0 (*t* = 0). In separate preparations, we tested whether a variety of drugs were applied during the dissection period and then washed out prior to recording; 3 μM indomethacin (a non-selective inhibitor of cyclooxygenases, *n* = 3); 10 μM ketotifen (a mast cell stabilizer, *n* = 3); or 10 μM forskolin (an adenylate cyclase activator, *n* = 3). All recordings were made in normal Krebs solution.

### Recordings with mucosa attached

Four preparations were studied under conditions where damage to the tissue was minimized by leaving the mucosa and submucosa attached to the circular muscle. Circular muscle cells were impaled through the serosa and longitudinal muscle layer and identified by the presence of characteristics large IJPs.

### Drugs

All drugs were purchased from Sigma Aldrich (Sydney, Australia). EDTA was stored at 10^−1^M in aqueous solution used at final concentration of 1 mM; indomethacin was dissolved in DMSO at 10^−2^M and used at final concentration of 3 μM; ketotifen was kept in aqueous solution at 10^−2^M and used at a final concentration 10 μM; forskolin was dissolved in DMSO at 10^−2^M and used at a final concentration of 10 μM.

### Data analysis

Results are expressed as means ± standard error of mean and “*n*” refers to the number of animals contributing to each observation. Tissue from a total of 41 animals was studied. Multiple preparations from the same animal were not used for the same experiment. Two-tailed *t*-tests were used to compare paired or unpaired data samples with care taken to avoid multiple comparisons. Linear regression analysis was used to quantify the development of responses over time. Two-way ANOVA was used to compare the recovery of responses over time between recording sites 1 and 4 mm from the edge (Bonferroni *post-hoc* test). One-Way ANOVA was used to compare the effects of varying degrees of stretch versus control, using PRISM software (Prism 4 for Macintosh, USA, 2003).

## Results

### Re-cutting edges of a responsive preparation

Three studies were carried out to determine the effects of cutting the edges of a preparation on junction potentials and dye coupling. First, 12 preparations were pinned out with a double row of pins around all 4 edges and allowed to equilibrate fully (120 min in warmed Krebs solution), recording cell responses. The 4 edges of the preparation, between the two rows of pins (approximately 1 mm wide), were then re-cut and cell responses were again measured. All recordings were made 2–3 mm from the original edge of the preparation. Since circular muscle cells are less than 500 μm long in these preparations(Carbone et al., 2012), this ensured that recordings were not made from cells that had been directly subjected to the cutting procedure.

**Figure 1:**
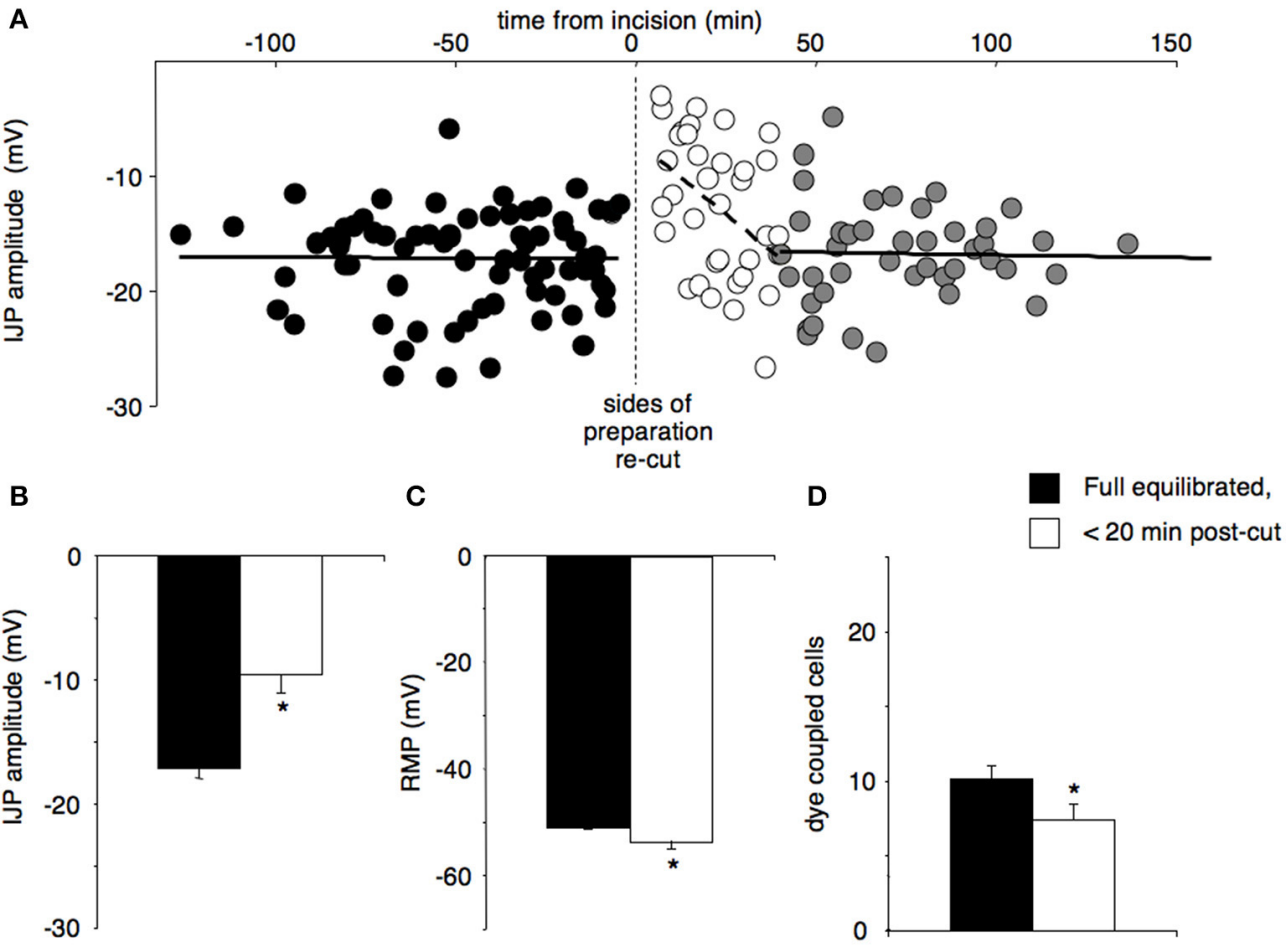
**The effects of re-cutting all four edges of a preparation, after full responses had recovered**. Preparations with double rows of pins were allowed to equilibrate fully, then approximately 0.5–1 mm of tissue between the rows of pins was re-cut, creating 4 new edges. **(A)** The amplitudes of IJPs were constant (17.2 ± 0.7 mV) before the incision (black circles, cells = 73, *n* = 12). The amplitude of IJPs was significantly reduced immediately following the cut (−9.6 ± 1.5 mV, open circles, *P* < 0.01, cells = 35, *n* = 12) and then recovered within approximately 40 min to the original value (gray circles; cells = 43, *n* = 12). **(B)** The amplitudes of IJPs were significantly reduced within 20 min after the cut compared to pre-cut (^*^P < 0.001, *n* = 12). **(C)** Resting membrane potential was significantly hyperpolarised after the cut versus pre-cut (^*^P < 0.05, *n* = 12). **(D)** The mean number of dye coupled cells was also significantly reduced after re-cutting the edges of the preparation (^*^P < 0.05, *n* = 12).

Re-cutting all four edges caused a significant reduction in fast IJP amplitude from −17.2 ± 0.7 to −9.6 ± 1.5 mV (*n* = 12, Figures 1A,B). Resting membrane potential hyperpolarised significantly (pre-cut = −50.6 ± 0.7 vs. post-cut = −53.5 ± 1.2, *P* < 0.05, *n* = 12, Figure 1C). Input resistance did not change significantly (6.1 ± 1.1 MΩ pre cut, 5.9 ± 0.9 MΩ post cut, *n* = 12), but re-cutting the edges significantly reduced the numbers of dye-coupled cells to 7.4 ± 1.0 cells from 10.1 ± 0.9 cells (*P* < 0.05, *n* = 12, Figure 1D). The fast response to exogenous ATP, which is largely due to excitation of inhibitory motor neurons (Carbone et al., 2012), reduced from 12.3 ± 1.3 mV before re-cutting, to 8.4 ± 1.3 mV after (*P* < 0.05, *n* = 12). The slow response to exogenous ATP, which is due to direct activation of P2Y1 receptors on the smooth muscle apparatus, was reduced from 11.8 ± 1.4 mV to 8.6 ± 1.3 mV. All of these changes replicate features of the initial equilibration period (Carbone et al., 2012). All of these responses then recovered over the ensuing 60 min to fully equilibrated values (Table 1).

**Table 1:** **The effects of re-cutting a preparation after recovery from dissection**.

	**Responsive preparation**	**<20 min of cutting preparation**
Fast IJP amplitude (mV)	−17.2 ± 0.7	−9.6 ± 1.5^ξ^
Fast ATP hyperpolarisation (mV)	−12.3 ± 1.3	−8.6 ± 1.4^*^
Slow ATP hyperpolarisation (mV)	−8.0 ± 0.7	−6.3 ± 0.7^§^
RMP (mV)	−50.6 ± 0.7	−53.5 ± 1.2^*^
Input resistance (MΩ)	6.1 ± 1.1	5.9 ± 0.9
Number of dye filled cells	10.1 ± 0.9	7.4 ± 1.1^*^

*Values represent means ± s.e.m., n = 12*,

* *P < 0.05*,

§ *P < 0.01*,

ξ *P < 0.0001*.

**Figure 2:**
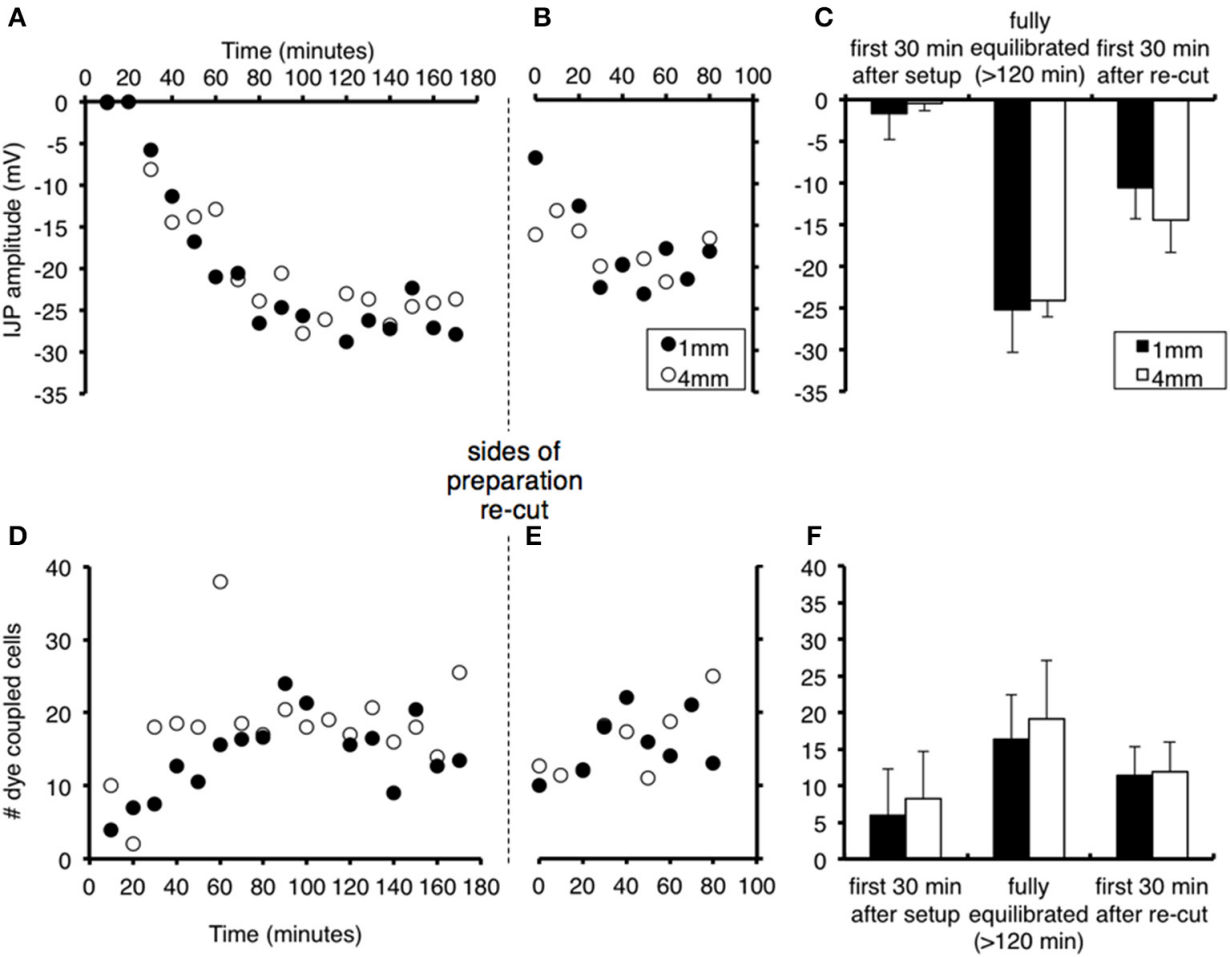
**Effects of proximity to the edge of the preparation on recovery of responses and effects of re-cutting the edges of the preparation**. Recordings were made alternately from smooth muscle cells 1 mm (filled circles) or 4 mm from the edge of the tissue (open circles) after initial set up and after re-cutting both sides of the preparation. Symbols represent averaged responses in 10 min bins, in order to display clearly the similar time courses for cells at 1 and 4 mm. **(A)** The IJP increased in amplitude from *t* = 0, with similar time course for cells 1 mm (black dots) or 4 mm (gray squares) from the edge of the preparation (*n* = 4). Edges were re-cut at the vertical dashed line. **(B)** IJPs were significantly reduced immediately after the cut, but not to the level after the initial setup. **(C)** The amplitude of IJPs (mean + standard deviation) was negligible immediately following dissection, and recovered after 120 min in cells at both 1 and 4 mm (averaged over 30 min periods). Mean responses decreased significantly after the edges were re-cut. There were no significant differences between cells 1 vs. 4 mm from the edge of the preparation at any point (*n* = 4). **(D–F)** Equivalent changes in numbers of dye coupled cells after standardized intracellular injection of carboxyfluorescein dye. Note the drop in coupling after the sides of the preparation were re-cut. These results show that both the initial set-up procedure and the act of cutting the edges of the preparation, affects cells at different distances equally.

In a second study, we investigated whether proximity of the recorded cell to the site of damage affected the magnitude of these effects. Cells either 1 or 4 mm from one side of the preparation were recorded alternately during the period after the initial setup, and after both sides had been re-cut (*n* = 4). Re-cutting both sides of the preparation (i.e., the edge running oral-anal) led to a significant reduction of IJP amplitudes at both 1 and 4 mm, although not as marked as the suppression that occurred at initial setup (cells at 1 mm declined from −25.2 ± 5.1 mV to −10.6 ± 3.7 mV (*P* < 0.01); cells at 4 mm declined from −24.1 ± 1.9 to −14.5 ± 3.9 mV (*P* < 0.01, see Figure 2). The IJPs then increased in amplitude over the next 80 min. In parallel, dye coupling was also slightly, but non-significantly reduced, at both sites, after re-cutting the sides of the preparation (1 mm decreased from 15.9 ± 4.3 cells to 11.3 ± 3.3 cells (*P* < 0.05); 4 mm decline from 18.8 ± 5.5 cells to 12.3 ± 4.0 cells).

**Figure 3:**
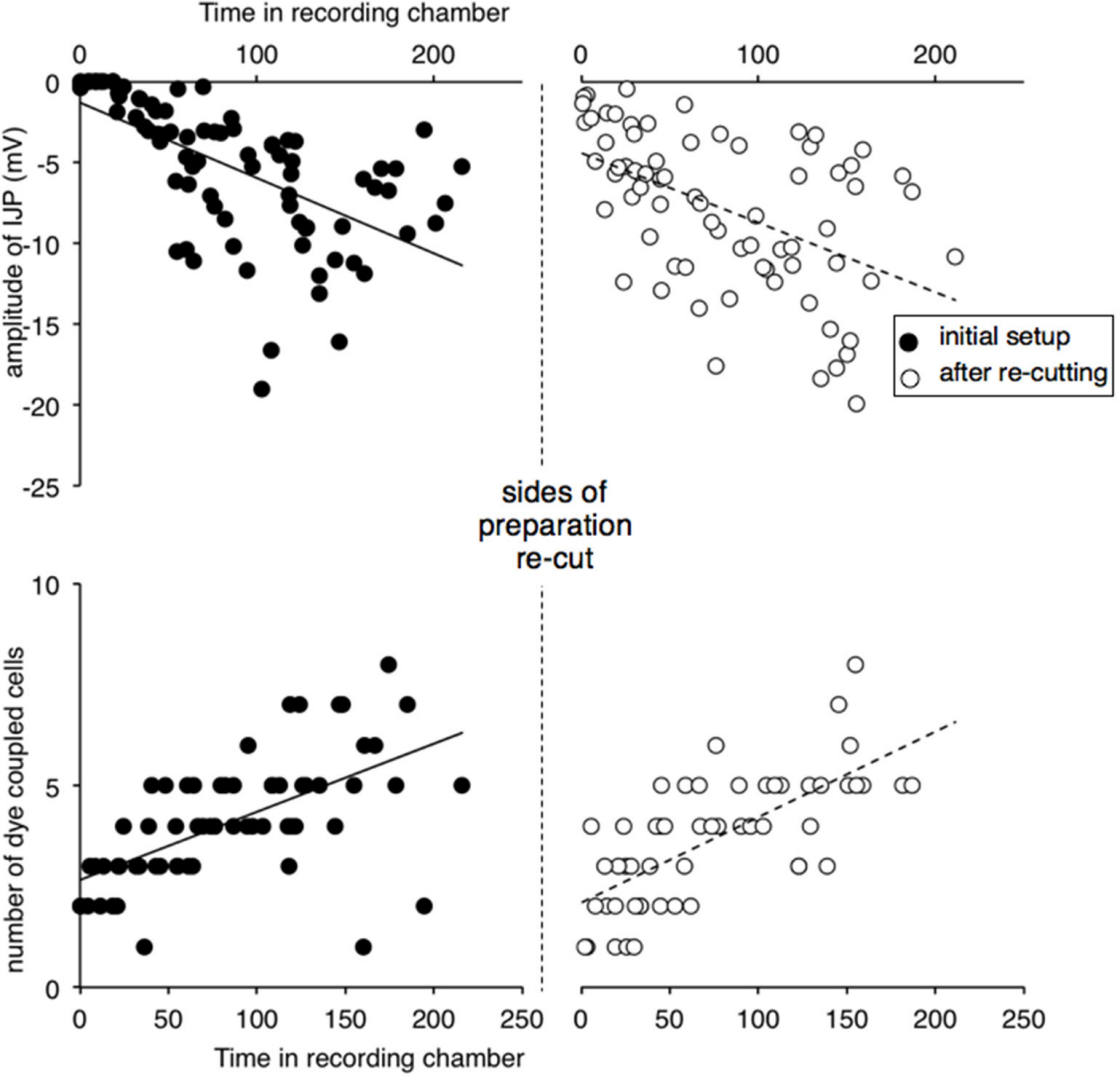
**Replication of effects of re-cutting the edges of preparation**. Recordings were carried out by an independent researcher. Preparations (*n* = 4) were recorded after initial set-up and after re-cutting the edges either side of the preparation. Inhibitory junction potentials were initially immeasurable, when preparations were first recorded and increased in amplitude over the following hours. Dye coupling, measured by spread of carboxyfluorescein injected from the recording electrode, also increased in parallel. After the edges of the preparation were re-cut (vertical dashed line) both IJP amplitude and dye coupling reduced significantly, recovering gradually with time.

Because of the importance of this result and variability in the data, the basic experiment was repeated by a different researcher who set up, recorded and analyzed the data independently. Preparations of guinea pig ileum were set up (*n* = 5) and the amplitude of IJPs and numbers of coupled cells monitored during the first 3 h. Both sides of the preparation were then re-cut and IJPs and coupled cells counted again over the ensuing 3 h. The results are shown in Figure 3. The major results were replicated: IJPs were initially suppressed and slowly recovered in amplitude, as occurred with dye coupling. Re-cutting the 2 sides of the preparation (that ran oral-anal) caused a significant reduction in IJP amplitude averaged over the 30 min period either side of the cut (from 8.1 ± 2.0 mV to 3.7 ± 2.0 mV, *t* = 2.834, *df* = 4, *P* < 0.05) and a temporary, but significant reduction in dye coupling (4.7 ± 0.9 to 2.2 ± 1.4 cells, *t* = 5.116, *df* = 4, *P* < 0.01), that again recovered slowly.

### Stretching a responsive preparation

Stretching preparations to by up to 150% had little effect on the responses recorded at resting circumference, in the first 20 min after the stretch (*n* = 4). The amplitude of fast IJPs; the amplitude of fast and slow ATP evoked hyperpolarisations; resting membrane potential, input impedance and the number of dye filled cells were not significantly different in control cells versus those impaled after stretching of up to 115, 130, and 150% of its resting length (Table 2). Stretches of 160% or more led to tearing of the preparation.

**Table 2:** **Stretching preparations had little effect on subsequent responses of smooth muscle cells recorded at resting length (*n* = 4)**.

	**Responses within 20 min of stretching**			
	**Resting length**	**115% stretch**	**130% stretch**	**150% stretch**
Fast IJP amplitude (mV)	23.7 ± 3.3	22.4 ± 2.9	16.4 ± 2.4	17.8 ± 2.4
Fast ATP hyperpolarisation (mV)	15.2 ± 4.1	15.4 ± 2.2	11.9 ± 0.9	14.4 ± 3.3
Slow ATP hyperpolarisation (mV)	8.1 ± 1.3	8.8 ± 1.8	8.2 ± 1.7	8.7 ± 1.5
RMP (mV)	−49.4 ± 1.5	−49.9 ± 0.7	−51.3 ± 2.0	−51.3 ± 1.7
Input resistance (MΩ)	7.6 ± 2.1	7.3 ± 0.5	6.5 ± 1.4	6.6 ± 1.3
Number of dye filled cells	10.3 ± 0.9	12 ± 1.5	8.9 ± 2.1	9.1 ± 2.2

### Limiting influx of Ca^2+^ from krebs solution during dissection

Four preparations were dissected in low Ca^2+^ (0.25 mM), high Mg^2+^ (10 mM) Krebs solution, to reduce Ca^2+^-influx to cells damaged during the set-up procedure. They were then superfused with normal warmed Krebs solution from *t* = 0. This did not block the development of an initial, unresponsive period; IJPs were initially suppressed, similar to preparations dissected in normal Krebs solution (*n* = 4). Mean fast IJP amplitude was −0.3 ± 0.3 mV in the first 30 min. This recovered partially to −8.4 ± 1.5 mV by 90–120 min (*n* = 4, *P* < 0.001). Mean number of dye coupled cells was significantly smaller in the first 30 min compared to 90–120 min (1.1 ± 0.1 versus 4.5 ± 0.5 versus *P* < 0.0001, *n* = 4). It should be noted that both IJP amplitude and dye coupling did not recover to normal levels within the 120 min, suggesting that prolonged exposure to the low [Ca^2+^] solution may have impaired cellular function in the preparations.

### Cyclo-oxygenase inhibitor effects

Preparations were also pre-incubated and dissected in indomethacin (3 μM). This was then washed out and the preparation was superfused with normal, warmed Krebs solution, from *t* = 0. The presence of indomethacin during dissection did not prevent the initial suppression of IJPs. Fast IJP amplitudes within the first 30 min averaged 0 ± 0.0 mV but significantly increased to −14.1 ± 1.0 mV (*P* < 0.001, *n* = 3) after 120 min, similar to preparations dissected in control Krebs solution. The amplitude of the fast ATP-evoked hyperpolarisation was negligible within the first 30 min and increased to −11.8 ± 1.3 mV after 120 min (*P* < 0.0001, *n* = 3). The slow ATP-evoked hyperpolarisation averaged −0.2 ± 0.4 mV in amplitude in cells impaled in the first 30 min; this increased to −7.4 ± 0.5 mV after 120 min (*P* < 0.001, *n* = 3). Resting membrane potential in the first 30 min was −64.1 ± 3.0 mV, which was significantly hyperpolarised compared to cells impaled after 120 min (−49.9 ± 0.5 mV, *P* < 0.05, *n* = 3). Mean input impedance showed a non-significant trend; cells impaled within the first 30 min had slightly greater input resistance than cells after 120 min (9.8 ± 4.1 vs. 5.3 ± 0.5 MΩ, *n* = 3). On average, 5.5 ± 1.5 cells were dye filled in the first 30 min, compared to 10.0 ± 0.8 cells filled after 120 min (*P* < 0.05, *n* = 3).

### Mast cell stabilizer effects

Pre-incubating and dissecting preparations in Krebs solution with the mast cell stabilizer ketotifen (10 μM) had no effect on the occurrence of an initial unresponsive period after set-up. Fast IJPs still had unmeasurable amplitudes in cells impaled in the first 30 min. Their amplitude increased significantly 120 min after dissection (from 0.0 ± 0 mV to 18.8 ± 2.4 mV, *n* = 3, *P* < 0.05). Fast and slow hyperpolarisations evoked by exogenous ATP were immeasurably small within the first 30 min, but after 120 min fast ATP responses averaged 10.8 ± 1.0 mV and slow ATP evoked responses were −6.5 ± 0.9 mV (*P* < 0.05, *n* = 3). Resting membrane potentials in the first min was significantly hyperpolarised compared to cells impaled after 120 min (−60.7 ± 2.6 mV to −48.4 ± 1.2 mV, *P* < 0.05, *n* = 3). Input resistance decreased after the first 30 min of dissection (14 ± 3.0 MΩ vs. 10.0 ± 3.1 but again this was not significant, *n* = 3). On average, 4.7 ± 2.7 cells were dye filled in the first 30 min compared to 7.8 ± 0.2 after 120 min (not significant, *n* = 3). Ketotifen pre-treatment did not block the occurrence of the initial unresponsive period.

### Forskolin effects

When preparations were pre-incubated and dissected initially in 10 μM forskolin, the unresponsive period still occurred. Fast IJP amplitude were unmeasurable in the first 30 min, but after 120 min averaged −5.9 ± 0.8 mV (*n* = 3). Fast and slow ATP-evoked hyperpolarisations were also too small to measure within 30 min of set-up, but after 120 min increased to −5.8 ± 3.0 mV and −4.1 ± 1.6 mV, respectively. The mean number of dye coupled cells in the first 30 min was 4.3 ± 1.9, compared to 8.7 ± 2.8 after 120 min (*n* = 3). These observations show that exposure to forskolin, which increases intracellular cyclic AMP concentrations, did not block the occurrence of the initial unresponsive period.

### Recordings in minimally dissected preparations (mucosa attached)

**Figure 4:**
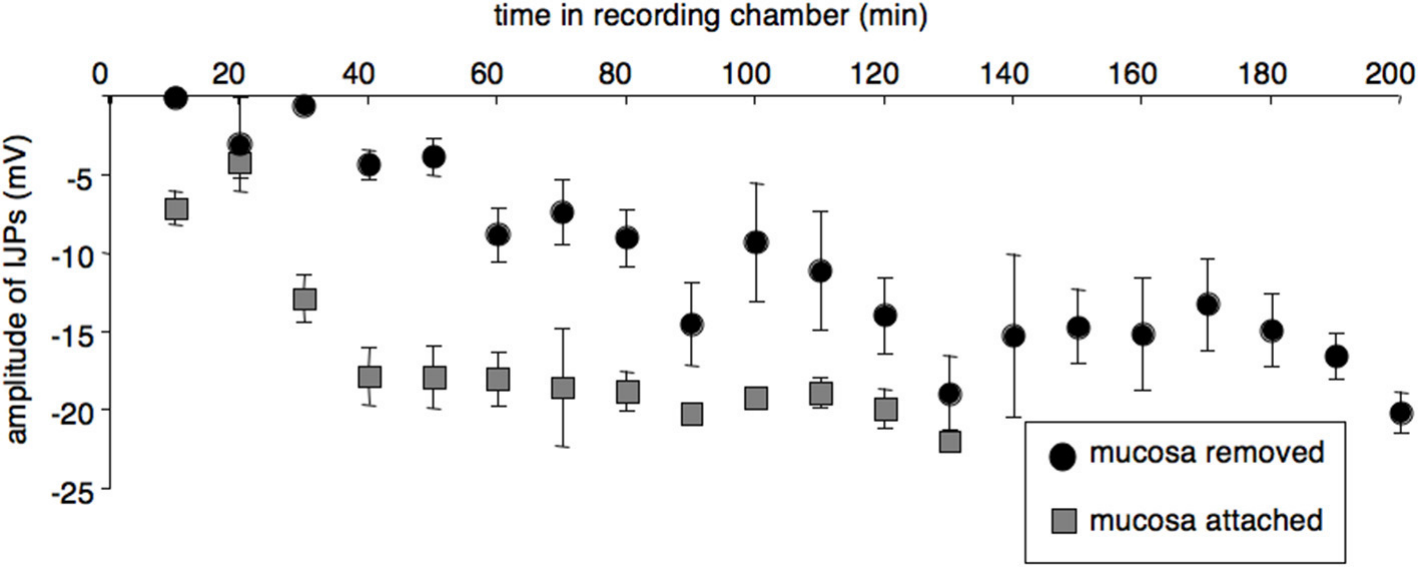
**Leaving the mucosa and submucosa attached reduced both the extent and duration of the initial impairment of smooth muscle function**. **(A)** IJPs from preparations with attached mucosa and submucosa (averaged in 10 min bins, gray squares; *n* = 4, cells = 41) were initially larger and recovered more rapidly after initial set-up than cells from preparations with mucosa and submucosa removed (black circles *n* = 4, cells = 33).

Four preparations were set up with the mucosa and submucosa left attached to the circular muscle layer (although they were opened up and pinned out as flat sheets, with the mucosa downwards). In these preparations, IJPs were initially reduced in amplitude, but not to the extent that occurred in preparations in which the mucosa and submucosa had been removed. In the first 30 min, IJPs averaged −8.8 ± 1.5 mV (*P* < 0.005, *n* = 4, cells = 10) compared to −0.0 ± 0.0 mV in preparations with mucosa and submucosa removed (*P* < 0.005, *n*= 4, 6 cells). In the partially dissected preparations, IJPs subsequently increased to a mean of 18.9 ± 0.7 mV (*P* < 0.005, *n* = 4, cells = 31, Figure 4). The initial suppression of responses was also of shorter duration than in fully dissected preparations in which the submucosa and mucosa had been removed. These results suggest that removal of the mucosa and submucosa exacerbated the initial loss of responses in the circular smooth muscle, but did not account fully for it. Resting membrane potential and input resistance in the first 30 min did not differ from fully dissected preparations. Dye coupling and ATP application could not be studied in these preparations due to high background fluorescence and opacity of the tissue.

## Discussion

It has previously been reported that responses in the smooth muscle of the guinea pig ileum are profoundly suppressed in the first 30 min after setting up *in vitro* preparations (Carbone et al., 2012). During this period there is a lack of muscle tone, spontaneous contractions are absent, responses to electrical stimulation and exogenous agonists are abolished and the preparation is apparently inactive. However, over the subsequent 30–90 min, physiological responses recover. Whilst this phenomenon is a minor inconvenience for physiologists and pharmacologists, if it were to operate *in vivo*, profound suppression of intestinal motility would ensue. Despite its potency, little is known about what causes the initial loss of responsiveness. This study has investigated which aspects of the set-up procedure are likely to contribute to this phenomenon.

In a previous study (Carbone et al., 2012) it was shown that the loss of responsiveness is not solely attributable to disruption of the contractile apparatus. Neuromuscular transmission, measured by electrical junction potentials, is also markedly suppressed. This is temporally associated with a loss of gap junction coupling between smooth muscle cells, quantified by dye coupling. As smooth muscle function spontaneously recovers, gap junction coupling also resumes, with a parallel time course (Carbone et al., 2012). Furthermore, when full responses have been attained, disruption of gap junctions (by carbenoxolone, 18β-glycyrrhetinic acid or 2-aminoethoxydiphenyl borate) causes loss of junction potentials and responses to agonists. This suggests that temporary loss of gap junction coupling, in the post-junctional smooth muscle apparatus may underlie the suppression of function in the first 30 min.

### Candidate mechanisms

The initial loss of coupling is likely to be due to one or more of the procedures that the tissue is subjected to during set-up. These include (i) removal of intestine and immersion in cooled Krebs solution (ii) cutting open the preparation along the mesenteric border and trimming it to length (iii) removal of the mucosa and submucosa which involves considerable stretch of the tissue, and (iv) pinning the preparation it in the recording chamber. After these steps, warmed Krebs solution was applied by superfusion. Any, or all, of these steps could have contributed to the initial loss of responses. In the previous study, it had been established that neuro-neuronal transmission was not equivalently impaired in the first 30 min (as measured by nicotinic fast excitatory post-synaptic potentials), suggesting that a generalized loss of neurotransmitter release from presynaptic/pre-junctional axons was unlikely. However, we cannot rule out the possibility that neurotransmitter release from motor neuron axons specifically, was affected by damage, in a manner that was not shared by other classes of enteric neurons.

### Damage as a cause of unresponsiveness

Two manipulations investigated in the present study modulated smooth muscle responses in a manner comparable to that seen in the first 30 min. First, re-cutting the edges of the preparation caused a marked reduction in both junction potential amplitude and dye-coupling which was comparable to, but of lesser amplitude and duration than the suppression that followed initial set-up. Importantly, these effects were not mediated by direct damage to recorded cells because recordings were always made more than 1 mm from the nearest edge (corresponding to more than 2 smooth muscle cell lengths). In addition, similar magnitudes of effect were seen at 1 and 4 mm from the cut edge.

A second contributor is also implicated. Full suppression of IJPs was seen in the first 30 min in preparations in which the mucosa and submucosa had been peeled off the circular muscle layer. However, the initial suppression was significantly less in preparations in which this step had been omitted. Removing the mucosa and submucosa must inevitably cause damage to superficial smooth muscle cells across the entire preparation. The extent of this damage was hard to measure directly, as it was not possible to remove this layer during recordings. However, the observation that less-traumatized preparations with mucosa and submucosa attached had significantly larger IJPs in the first 30 min is strong support for the proposal that tissue damage contributes to the loss of coupling and suppression of smooth muscle responsiveness. Peeling the submucosa from the circular muscle layer inevitably stretches the smooth muscle. The present study suggests that stretch, *per se*, is unlikely to be the cause of impaired function. Rather, it seems more likely that the shear forces and physical trauma to the surface of the circular muscle layer is one of the culprits. Additional damage caused by the initial opening of the preparation and cutting to size also clearly contributed, as shown by the effects of re-cutting the edges.

### How damage may affect smooth muscle physiology

We speculate that cutting the edges, and peeling off the submucosa cause changes in intracellular contents of damaged smooth muscle cells. These effects then spread rapidly through the tissue, possibly via intercellular junctions. Cytosolic Ca^2+^ concentration can modulate gap junction conductance, via a calmodulin dependent protein kinase (Bloomfield and Völgyi, 2009). In addition, smooth muscle cells express several types of Ca^2+^ dependent K^+^ channels (Vogalis, 2000) which could contribute to the initial hyperpolarisation of unresponsive smooth muscle cells. Dissecting and setting up preparations in low calcium-solution did not prevent the initial suppression of responses. However, calcium ions were still present at an extracellular concentration of 250 μM in this solution, which is 3–4 orders of magnitude higher than intracellular level. Thus, we cannot rule out the possibility of a significant rise in [Ca^2+^]_i_ in smooth muscle cells damaged during set-up. Protons are also acutely released on cell damage (Woo et al., 2004) and pH modulates gap junction coupling between ICC (Belzer et al., 2004), and between smooth muscle cells in the guinea pig ileum (Kobilo et al., 2003). Intracellular magnesium ion concentrations can decrease gap junction coupling in the heart (Matsuda et al., 2010). The mechanisms that cause the initial loss of dye coupling and the reduction in IJPs remain to be determined. Nevertheless, the present study suggests that the mechanisms are likely to be triggered by acute damage to the intestinal wall; probably the smooth muscle apparatus itself.

### Role of soluble mediators

It is theoretically possible that soluble mediators, diffusing from damaged cells, may contribute to suppression of smooth muscle function. Mast cells are present in the submucosa and at the border of the circular muscle (Mota et al., 1956) and would unavoidably be affected by the dissection procedure. However, pre-treatment with the mast cell stabilizer, ketotifen, did not block the initial suppression of smooth muscle responses. Pre-incubation and dissection in indomethacin, a potent blocker of cyclo-oxygenases, also failed to reduce the unresponsive period, suggesting that prostaglandin release was not an essential player. ATP is a potent mediator that is released by damaged cells (Cook and Mccleskey, 2002), but in the present study ATP was repeatedly applied to smooth muscle cells without causing any detectable loss of coupling.

### Other inflammatory mechanisms

Damage to cells anywhere in the body activates a cascade of inflammatory events involving white blood cells, which release a mixture of mediators leading to vasodilation, plasma extravasation, sensitisation of primary afferent nerve endings and sympathetic activation. These mechanisms have been extensively investigated in post-operative ileus (Boeckxstaens and De Jonge, 2009). The initial stage occurs during the course of surgery, and is followed by a more protracted series of neuro-immune interactions initiated by mast cells and resident macrophages. The present study has demonstrated that direct damage of the gut wall can activate reversible mechanisms that can profoundly impair smooth muscle contractility. They require neither extrinsic neural pathways, nor an intact blood supply to operate. If these mechanisms are activated during normal physiology, or under pathophysiological conditions, they may potently modulate smooth muscle function.

## Author contributions

Simona E. Carbone and Timothy J. Hibberd contributed to the experimental design, data gathering and analysis, drafting and revision of manuscript. Simon J. H. Brookes, David A. Wattchow, and Nick J. Spencer contributed to conception of the project, experimental design and revision of manuscript.

### Conflict of interest statement

The authors declare that the research was conducted in the absence of any commercial or financial relationships that could be construed as a potential conflict of interest.
